# Effectiveness of the autumn 2023 COVID-19 vaccine dose in hospital-based healthcare workers: results of the VEBIS healthcare worker vaccine effectiveness cohort study, seven European countries, season 2023/24

**DOI:** 10.2807/1560-7917.ES.2024.29.44.2400680

**Published:** 2024-10-31

**Authors:** Camelia Savulescu, Albert Prats-Uribe, Kim Brolin, Anneli Uusküla, Colm Bergin, Catherine Fleming, Rita Murri, Viesturs Zvirbulis, Dace Zavadska, Vania Gaio, Corneliu P Popescu, Raluca Hrisca, Maria Cisneros, Miriam Latorre-Millán, Liis Lohur, Jonathan McGrath, Lauren Ferguson, Katleen De Gaetano Donati, Ilze Abolina, Dagne Gravele, Ausenda Machado, Simin-Aysel Florescu, Mihaela Lazar, Pilar Subirats, Laura Clusa Cuesta, Jacklyn Sui, Claire Kenny, Rosaria Santangelo, Dainis Krievins, Elza Anna Barzdina, Camila Valadas Henriques, Alma Gabriela Kosa, Saftica-Mariana Pohrib, Carmen Muñoz-Almagro, Ana Milagro, Sabrina Bacci, Anthony Nardone, VEBIS HCW VE study group, Collaborators in VEBIS HCW study group, Hanna Sepp, Lisa Domegan, Joan O’Donell, Maeve Kernan, Anna Moriarty, Conor Moran, Giulia De Angelis, Silvia Lamonica, Giulia Menchinelli, Cinzia Recine, Silvia Zelli, Alice Tondinelli, Benedetta Falasca, Janis Meisters, Laura Zemite, Hilda Darta Snipe, Estere Ergle, Ana Palmira Amaral, João Almeida Santos, Daniela Dias, Licinia Gomes, Miguel Lança, Henrique Carvalho, Jose Alves, Daniel R. Codreanu, Alexandru Marin, Amaresh Pérez-Arguello, Iolanda Jordan, Juan Jose Garcia-Garcia, Antonio Rezusta, Alexander Tristancho, Ignacio Ezpeleta, Sandra Dueñas, Noelia Terren, Tamara Valero, Nieves Felisa Martínez, Yolanda Gracia, David Martínez, Beatriz Gilaberte, Cristina Carrasco, Carmen Martínez, Maria Pilar Rubio, Maria Gemma Martínez, Bruno del Moral, Ana Maria Sabio, Raquel Guiomar, Aryse Melo, Francisco Pozo, Ranya Mulchandani, Madelyn Rojas

**Affiliations:** 1 Epiconcept, Paris, France; 2 These authors contributed equally to this work and share first authorship. The other co-authors are listed in the alphabetical order of their countries.; 3 European Centre for Disease Prevention and Control, Stockholm, Sweden; 4 University of Tartu, Institute of Family Medicine and Public Health, Tartu, Estonia; 5 Saint James’s Hospital, Dublin, Ireland; 6 Galway University Hospital, Galway, Ireland; 7 Fondazione Policlinico Universitario A, Gemelli IRCCS, Rome, Italy; 8 Università Cattolica del Sacro Cuore, Rome, Italy; 9 Pauls Stradins Clinical University Hospital, Riga, Latvia; 10 Children Clinical University Hospital, Riga, Latvia; 11 Department of Epidemiology, National Institute of Health Doutor Ricardo Jorge, Lisbon, Portugal; 12 Victor Babes Clinical Hospital of Infectious and Tropical Diseases, Bucharest, Romania; 13 Carol Davila University of Medicine and Pharmacy, Bucharest, Romania; 14 Central Military Emergency University Hospital “Dr. Carol Davila”, Bucharest, Romania; 15 Institut de Recerca Sant Joan de Deu, Hospital Sant Joan de Deu, Barcelona, Spain; 16 Medicine Department, Universitat Internacional de Catalunya, Barcelona, Spain; 17 Research Group on Difficult to Diagnose and Treat Infections, Miguel Servet University Hospital, IIS Aragon, Zaragoza, Spain; 18 Viljandi Hospital, Viljandi, Estonia; 19 Cantacuzino National Military-Medical Institute for Research and Development, Bucharest, Romania; 20 Department of Occupational Risk Prevention, Hospital Sant Joan de Deu, Barcelona, Spain; 21 Department of Infectious Diseases, National Institute of Health Doutor Ricardo Jorge, Lisbon, Portugal; 22 Ciber of Epidemiology and Public Health CIBERESP, Madrid, Spain; 23 The additional collaborators of the VEBIS HCW VE study group are listed at the end of the article.

**Keywords:** Vaccine effectiveness, COVID-19, SARS-CoV-2, Healthcare workers, Europe

## Abstract

COVID-19 vaccination recommendations include healthcare workers (HCWs). We measured COVID-19 vaccine effectiveness (CVE) of the autumn 2023 dose against laboratory-confirmed SARS-CoV-2 infection in a prospective cohort study of 1,305 HCWs from 13 European hospitals. Overall CVE was 22% (95% CI: −17 to 48), 49% (95% CI: −8 to 76) before and −11% (95% CI: −84 to 34) after the start of BA.2.86/JN.1 predominant circulation. Autumn 2023 COVID-19 vaccination led to a moderate-to-low reduction in SARS-CoV-2 infection incidence in HCWs. Monitoring of CVE is crucial for COVID-19 prevention.

COVID-19 vaccination recommendations prioritise healthcare workers (HCWs), considering their exposure to severe acute respiratory coronavirus 2 (SARS-CoV-2) and their key role in the functioning of healthcare systems. In the European Union/European Economic Area (EU/EEA), HCWs were considered a priority for COVID-19 revaccination during the autumn 2023 campaign [1], and the World Health Organization (WHO) recommended revaccination of HCWs 12 months after their last dose [2]. Because the Omicron sub-lineage XBB.1.5 predominated in spring 2023, the COVID-19 vaccines were adapted to target this emerging strain, and the first XBB.1.5 vaccine was authorised for use in the EU/EEA in August 2023. Omicron BA.2.86/JN.1 emerged in the EU/EEA at the end of 2023, according to data available on the European Respiratory Virus Surveillance Summary (ERVISS) [3]. Evidence for COVID-19 vaccine recommendation in the HCW population remains scarce. Within the Vaccine Effectiveness, Burden and Impact (VEBIS) project, we aimed to measure the COVID-19 vaccine effectiveness (CVE) in HCWs, in the winter season 2023/24. 

## VEBIS healthcare worker cohort

In this prospective cohort study [4], we recruited HCWs from 13 hospitals in seven countries (Estonia, Ireland, Italy, Latvia, Portugal, Romania, and Spain). At a weekly follow-up, HCWs provided nasopharyngeal or saliva samples to detect incident SARS-CoV-2 infections and completed a questionnaire to update vaccination and exposure information. We excluded HCWs who did not provide informed consent, missed important information for analysis (e.g. vaccination status and laboratory results) or presented discordant serology and virology results.

## Definition of exposures, outcomes, covariates

We defined current vaccination as HCWs who received a dose of any COVID-19 vaccine brand during the autumn 2023 campaign and unvaccinated as HCWs who did not receive a vaccine dose during this campaign, regardless of the number of doses and timing of previous vaccination(s). We stratified previous vaccination in (i) more than 365 days or unvaccinated before the autumn 2023 vaccination campaign and (ii) 90–365 days before the autumn 2023 campaign. We grouped the time since current vaccination in 7–59, 60–119 and ≥ 120 days.

The main outcome of the study was time to the first incident SARS-CoV-2 infection, detected by RT-PCR, regardless of symptoms. Secondary outcomes included symptomatic and asymptomatic COVID-19 in HCWs, depending on whether or not symptoms were reported from 14 days before to 7 days after the first positive test.

Recent previous SARS-CoV-2 infection was defined as self-reported SARS-CoV-2 infection after 1 November 2022 (the month with the start of predominant circulation of Omicron XBB sub-lineage in the participating countries). Non-recent previous infection was defined as self-reported previous SARS-CoV-2 infection before 1 November 2022. We excluded from all analyses a period of 60 days after a positive RT-PCR sample [5].

## Vaccine effectiveness analysis

First, we measured the CVE of the autumn 2023 vaccine dose, comparing the current vaccinated with unvaccinated HCWs. In secondary analyses, we measured the CVE by time since previous vaccination, by time since current vaccination, by recent previous infection overall, and by symptomatic status of the SARS-CoV-2 infection, stratified before and after the start of predominant circulation of the Omicron BA.2.86/JN.1 virus sub-lineage. Using Cox regression, we calculated effectiveness as:

CVE = (1 − hazard ratio of current vaccination) × 100.

We adjusted the CVE for hospital, age, sex, at least one underlying condition, and recent SARS-CoV-2 infection.

## Descriptive and vaccine effectiveness results

**Figure 1:**
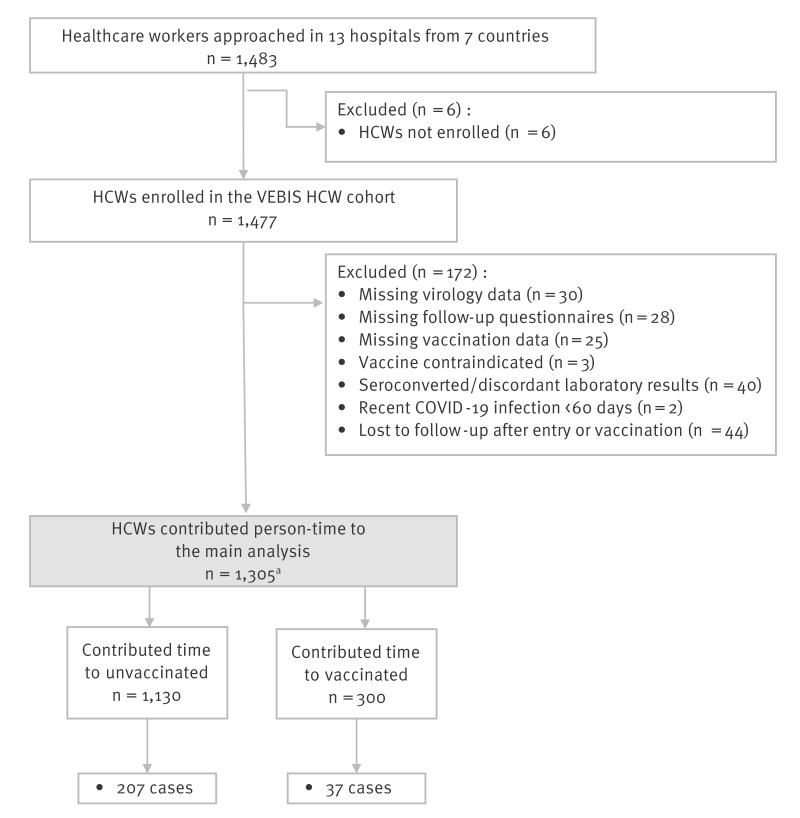
Inclusion and exclusion criteria, VEBIS healthcare worker multicentre cohort study on COVID-19 vaccine effectiveness, seven European countries, season 2023/24 (n = 1,483)

Between October 2023 and May 2024, out of 1,483 HCWs approached, 1,477 were enrolled, ranging from 160 in Italy to 304 in Romania. After applying the inclusion and exclusion criteria, 1,305 HCWs remained in the CVE analysis (Figure 1). Vaccinated HCWs were more likely to be older or to work as medical doctors, and less likely to be female or current smokers (Table 1).

HCW: healthcare worker.

^a^ HCWs could contribute to both vaccinated and unvaccinated groups.

**Table 1:** Main characteristics of participants at enrolment by vaccination status, VEBIS healthcare worker multicentre cohort study on COVID-19 vaccine effectiveness, seven European countries, season 2023/24 (n = 1,305)

Characteristic	Vaccinated in season 2023/24 (n = 300)		Not vaccinated in season 2023/24 (n = 1,005)	
	n	%	n	%
Sex				
Female	235	78.3	849	84.5
Male	65	21.7	156	15.5
Age group (years)				
18–34	40	13.3	224	22.3
35–39	33	11.0	111	11.1
40–44	47	15.7	143	14.2
45–49	52	17.3	177	17.6
50–54	42	14.0	154	15.3
≥ 55	86	28.7	196	19.5
Role				
Medical doctor	53	17.7	161	16.0
Nurse	74	24.7	491	48.9
Administration/reception	48	16.0	134	13.3
Ancillary	8	2.7	40	4.0
Allied	10	3.3	23	2.3
Laboratory	13	4.3	47	4.7
Other	38	12.7	98	9.8
Missing	56	18.7	11	1.1
Smoking				
Never smoked	170	56.7	509	50.6
Ex-smoker	70	23.3	255	25.4
Current smoker	32	10.7	234	23.3
Missing	28	9.3	7	0.7
Underlying conditions				
At least one	78	26.0	278	27.7
No underlying condition	212	70.7	698	69.4
Missing	10	3.3	29	2.9
Recent previous COVID-19 episode				
Yes	72	24.0	324	32.2
No	184	61.3	653	65.0
Missing	44	14.7	28	2.8
Time since last previous COVID-19 episode				
Median time in days (range)	521 (60–1371)		493 (60–1,481)	
Brand autumn vaccination dose				
Not XBB1.5-adapted	55	18.3	Not applicable	
XBB1.5-adapted	245	81.6		
Time since last vaccination dose				
Median time since previous dose in days (range)	895 (224–1,122)		741 (132–1,107)	
Number of vaccine doses ever received before the autumn 2023 vaccination campaign				
Unvaccinated	0	0.0	58	5.8
1 dose	2	0.7	44	4.4
2 doses	7	2.3	216	21.5
3 doses	38	12.7	502	49.9
4 doses	134	44.7	183	18.2
5 doses	119	39.7	2	0.2

**Figure 2:**
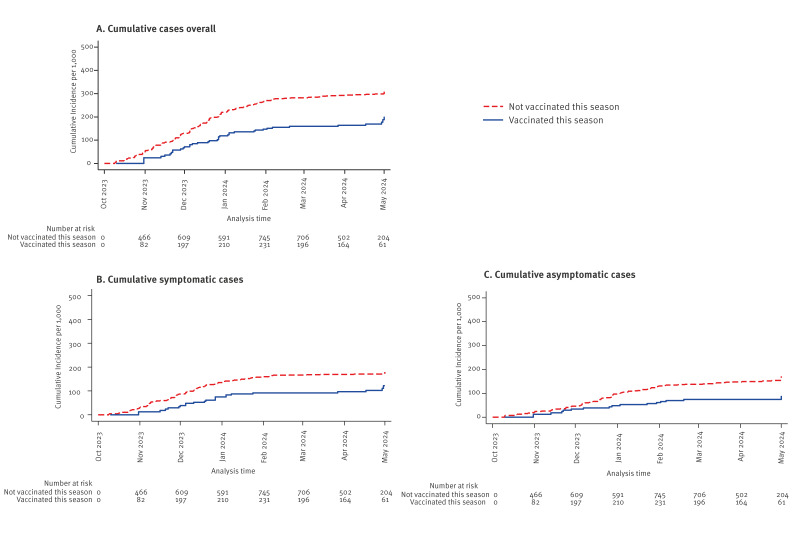
Kaplan-Meier plots of time (days) from enrolment to SARS-CoV-2 infection in VEBIS healthcare worker multicentre cohort study on COVID-19 vaccine effectiveness, by vaccination status, seven European countries, season 2023/24 (n = 1,305)

We detected 244 SARS-CoV-2 infections (Figure 1): 37 among vaccinated (1.03 per 1,000 person-days of observation) and 207 among unvaccinated HCW (1.7 per 1,000 person-days). Of these infections, 128 (52%) were symptomatic. The cumulative incidence was lower among the vaccinated throughout the entire follow-up regardless of the outcome used (Figure 2).

The adjusted CVE against SARS-CoV-2 infection was 22% (95% confidence interval (CI): −17 to 58) overall, with a CVE point estimate of 26% against asymptomatic and 17% against symptomatic infection, with overlapping confidence intervals. The CVE point estimates were 33% in HCWs with no recent prior infection and 23% in HCWs with a previous vaccination > 365 days. The CVE was 49% (95% CI: −8 to 76) before the start of BA2.86/JN1 circulation and below 0 during the BA2.86/JN1 circulation, with higher CVE point estimates 7–59 days after vaccination. The CVE point estimates were below 0 in HCWs with recent prior infection and vaccinated 90–365 days before the autumn dose, as well as ≥ 120 days after vaccination (Table 2).

**Table 2:** Adjusted vaccine effectiveness in the primary and secondary analyses, VEBIS healthcare worker multicentre cohort study on COVID-19 vaccine effectiveness, seven European countries, season 2023/24 (n = 1,305)

Analysis	Vaccinated			Unvaccinated			Adjusted^a^ CVE
	Number HCWs	Events	Person-days	Number HCWs	Events	Person-days	
Overall effect							
SARS-CoV-2 infection	300	37	35,657	1,130	207	121,394	22 (−17 to 48)
Asymptomatic infection	300	15	35,657	1,130	101	121,394	26 (−43 to 61)
Symptomatic COVID-19	300	22	35,657	1,130	106	121,394	17 (−40 to 51)
By recent prior infection							
No recent prior infection	184	25	21,135	738	150	70,024	33 (−9 to 58)
Recent prior infection	92	8	9,848	486	53	48,601	−9 (−139 to 50)
Time since previous vaccination							
Vaccinated 90–365 days before the current season vaccination	300	37	35,657	165	10	5,186	−31 (−245 to 50)
Vaccinated > 365 days before the current season vaccination	300	37	35,657	1,082	197	116,208	23 (−16 to 49)
Before/after BA.2.86 predominant circulation and time since vaccination in season							
**Before**	**190**	**9**	**4,999**	**871**	**108**	**36,503**	**49 (−8 to 76)**
7–59 days vs > 365 days before	190	9	4,854	801	100	32,278	47 (−11 to 75)
≥ 60 days vs > 365 days before	21	0	145	801	100	32,278	Not calculated
**After**	**296**	**28**	**30,658**	**963**	**99**	**84,891**	**−11 (−84 to 34)**
7–59 days vs > 365 days before	231	9	5,849	958	97	83,930	16 (−88 to 63)
≥ 60 days vs > 365 days before	283	19	24,809	958	97	83,930	−25 (−128 to 31)
Time since vaccination in season vs > 365 days before							
7–59 days	267	18	10,703	1,082	197	116,208	24 (−29 to 55)
60–119 days	251	9	6,658	1,082	197	116,208	39 (−43 to 74)
120–149 days	243	9	16,080	1,082	197	116,208	−2 (−114 to 52)

CVE: COVID-19 vaccine effectiveness; HCW: healthcare worker; SARS-CoV-2: severe acute respiratory syndrome coronavirus 2.

^a^ Adjusted by age sex, site, at least one underlying condition, recent previous SARS-CoV-2 infection (Participants with at least one covariate missing were not included in the adjusted model).

## Discussion

We estimated the effectiveness of an autumn 2023 COVID-19 vaccine dose in HCWs from 13 European hospitals of the VEBIS HCW prospective cohort study. The results suggest a moderate-to-low CVE among HCWs against SARS-CoV-2 infection overall. However, the CVE point estimates suggest a higher protection of COVID-19 vaccines against XBB.1.5 sub-lineages, in circulation before the start of predominant circulation of Omicron BA.2.86/JN.1. The CVE point estimates were higher in HCWs with non-recent previous SARS-CoV-2 infection, suggesting higher benefit of vaccination in these HCWs. The CVE point estimates were also higher for recent vaccination (< 60 days), even during predominant circulation of BA2.86/JN1 when CVE became lower than 0 after 60 days. The null CVE and wide confidence intervals in those with a recent prior vaccination suggest that, in the described scenario, vaccination more frequently than annually may not provide additional protection against SARS-CoV-2 infection overall.

Our results were similar to those of a study conducted in the United States in HCWs with similar vaccination coverage before (CVE = 42%) and during JN1 circulation (CVE = 19%) [6]. They were also similar to the overall estimates by time since vaccination during a 6-month season 2023/24 and to the estimates in HCWs with no recent previous infection in a study from the United Kingdom with higher vaccination coverage [7].

Disentangling the effect of time since last vaccination from the effect of virus evolution was of particular importance for CVE studies during the 2023/24 season [8,9]. As the protection remains at moderate level for about 4 months after vaccination and SARS-CoV-2 variants and sub-lineages continuously emerge, efforts need to be made to better predict the immune evasion [10] and take into account the antigenic distance in CVE estimation [11]. Meanwhile it remains necessary, in addition to vaccination, to recommend frequent testing for HCWs in contact with suspected cases in hospital and in the community, and to regularly reinforce the use of protective equipment when in contact with vulnerable patients, especially when new virus strains emerge.

One of the main strengths of our study was the frequent testing regardless of symptoms, which captured asymptomatic and milder infections; this is important in studies on emerging variants/sub-lineages and in the HCW population. Another strength was the thorough collection of vaccination and previous SARS-CoV-2 infection status.

The main limitation of the study was its low precision of CVE estimates, due to low uptake of the vaccine at the participating hospitals and to the limited number of events, resulting in small sample size particularly when further adjusting by other confounders such as the number of previous vaccine doses or professional role. Adding these covariates in the regression model increased the overall CVE point estimate by 8%, but with a poorer fit of the data than the reported model and with concerns around corelation with existing covariates in the model. Secondly, vaccinated participants seemed to be more likely to accept further vaccination: 45% had four doses compared with 18% in those unvaccinated during the 2023 vaccination campaign, potentially overestimating the CVE results (which was not the case when adjusting by number of previous vaccination doses, as described above). Finally, the studied season was characterised by an initial circulation of Omicron XBB.1.5, later replaced by sub-lineage BA.2.86 and its offspring JN.1. As eight hospitals lacked sequencing information to more accurately define the periods with predominant circulation of the Omicron sub-lineages, we used ERVISS data reported at country level as a proxy; further investigation is needed to check the consistency of our approach.

## Conclusion

Our results indicate that an autumn 2023 COVID-19 vaccine dose presented a moderate-to-low reduction of 22% in the risk of SARS-CoV-2 infection in HCWs overall. Nevertheless, the vaccine protected almost one in two HCWs in the period before the predominant circulation of BA.2.86/JN.1 sub-lineage and during less than 60 days after vaccination. Timely deployment of vaccines is crucial for the COVID-19 vaccination programme. With increased sample size, our VEBIS HCW cohort study can provide more precise information to inform key vaccination policies and public health interventions for HCWs in the following seasons.
